# EnzymeSifter: a tool for discovery of industrial enzymes from metagenomes

**DOI:** 10.1093/bioadv/vbag262

**Published:** 2026-09-03

**Authors:** Omar Darawsheh, Matthew Bashton

**Affiliations:** School of Geography and Natural Sciences, Faculty of Science and Engineering, Northumbria University, Newcastle upon Tyne, NE1 8ST, United Kingdom; School of Geography and Natural Sciences, Faculty of Science and Engineering, Northumbria University, Newcastle upon Tyne, NE1 8ST, United Kingdom

## Abstract

**Summary:**

Metagenomes contain vast amounts of sequences, complicating the process of identifying candidate enzymes for industrial applications. Industrial applications require evaluating multiple biochemical properties simultaneously, including solubility, thermal stability, and pH. While separate predictors for each property exist, a score that combines multiple predicted values will be more descriptive than those generated individually by distinct tools. We present EnzymeSifter, a tool that automates enzyme discovery from vast metagenomes and enables multi-property predictions. It identifies the best performing enzymes using a computed composite score of all predicted values and generates a phylogenetic tree to select the top candidate from each clade—ensuring diversity and even sampling of sequence space. It acts as a sieve that filters according to the user inputs and keeps the most promising non-redundant enzymes for experimental validation.

**Availability and implementation:**

EnzymeSifter is freely available and released under an MIT licence. EnzymeSifter source code is available at https://github.com/Bashton-Lab/EnzymeSifter

## 1 Introduction

The rapid development of metagenome analysis tools and wide availability of high throughput sequencing has substantially contributed to the growth in environmentally derived sequence data. These approaches yield massive numbers of candidate protein sequences, many of which may have applications as industrial enzymes, yet most of them will go untested experimentally. Hence, computational annotation methods used to screen enzyme performance under industrial conditions are of utmost importance to industry, as they reduce the time, effort, and cost needed for experimental validation. Some prediction methods can achieve high accuracy regarding the particular property they predict. However, to sufficiently evaluate the predicted enzyme performance, multiple properties must be considered. Necessitating a composite score that comprises various values to provide a comprehensive assessing approach.

We present EnzymeSifter, a tool that identifies enzymes from metagenomes, filters them according to user specified inputs, computing composite scores for the filtered enzymes considering all predicted values chosen by the user, and generates a neighbor-joining tree to mark the enzymes with the highest scores as representatives for their clades. This provides a standardised framework that reduces the effort required to go from large sequence datasets to a shortlist of promising enzymes that can be experimentally validated in the lab.

Generally, enzyme mining tools can be divided into reaction-based and property-based tools. Unlike reaction-based approaches, EnzymeSifter does not require a target reaction as it prioritises candidates from the user’s sequences based on their own desired predicted properties by selecting one or more EC numbers or Pfam families. Compared to EnzymeMiner 2.0 which is also a property-based tool (Rosinska *et al.* 2026), EnzymeSifter can be installed and run locally rather than being web server based, utilizes structural information, and performs no homology search, instead operates directly on the user’s own submitted sequences and their predicted structures.

## 2 Methods

### 2.1 Overview

EnzymeSifter is implemented in Python and makes use of Snakemake for scheduling the execution of external tools (Mölder *et al.* 2025). The first stage requires the path to FASTA sequences, users can optionally filter by the presence of certain motif(s), one or more Pfam families searched with HMMER 3.4 (Eddy 2011, Mistry *et al.* 2021), any level of the enzyme commission (EC) number(s) predicted using CLEAN (Yu *et al.* 2023), and/or sequence identity to cluster at with MMseqs2 (Steinegger and Söding 2017). The purpose of this stage is to reduce redundancy and lower the number of sequences. It writes a non-redundant FASTA of the retained sequences (*nonredundant.fasta*), thereby facilitating a reduced number of structural predictions required for stage two.

The second stage takes the path to the predicted PDB structures, and filters to keep only the structures with valid catalytic sites (*all_hits.fasta*) identified with EnzyMM (Hackett *et al.* 2026). The tool outputs the predicted values of solubility and usability using NetSolP—1.0 (Thumuluri *et al.* 2022), catalytic pH optimum pH_opt_ using pHoptNN (SinhaRoy *et al.* 2026), and thermal properties (enzyme optimal temperature *T*_opt_ and melting temperature *T_m_*) using Seq2Topt (Qiu *et al.* 2025) in (*all_predictions.tsv*). These tools are chosen based on their accuracy, speed, minimal dependencies, availability to install, and no limitations on enzyme class, see Supplementary Note S1.

Users have the option to filter the predicted properties to specific cutoffs for solubility and usability or intervals for pH_opt_ and *T*_opt_, *T*_m_ accepts both options to meet the user needs (*all_predictions_filtered.tsv*). MUSCLE v5.3 is used to align the filtered enzyme sequences (*muscle.afa*) (Edgar 2022), a neighbor-joining tree is then constructed with Biopython (*nj_tree.nwk*) (Saitou and Nei 1987, Cock *et al.* 2009), BLOSUM62 substitution matrix is used to compute pairwise distances (Henikoff and Henikoff 1992). The tree can be partitioned into a user-defined number of clades with a best case representative for each clade selected based on the user’s filtering criteria (*nj_tree_clades.png*). The selected representative is the enzyme with the highest computed composite score within its clade that passes all user supplied filters (*clade_representatives.tsv*). By enabling the user to stipulate a defined number of clades within the tree, they can dictate the number of best in clade candidate enzymes that can be screened in the lab with the automatically chosen representatives representing the most promising candidates within each clade. A flowchart of EnzymeSifter is provided at Fig. S1.

### 2.2 Testing EnzymeSifter

#### 2.2.1 First stage

To demonstrate the functionality of our tool, publicly available sequences were obtained from MGnify (pipeline v5.0, MGYS00006774 study) (Zhou *et al.* 2020, Richardson *et al.* 2023), the metagenomic reads were from 28 soil samples and contained more than 2.3 million protein sequences. For this test run the target enzymes were trypsins, all the sequences were filtered to keep only those with the GDSGGP motif, a highly conserved motif in trypsin (Lazarević and Janković‐Tomanić 2015), then by the trypsin Pfam family (PF00089), and were clustered at 90% sequence identity. This resulted in 166 non-redundant trypsin sequences. The 166 structures were predicted using the AlphaFold server (AlphaFold 3) (Abramson *et al.* 2024), with default settings. The output model_0 CIF file from each predicted structure was converted to the PDB format using GEMMI (Wojdyr 2022), without modifying the coordinates.

#### 2.2.2 Second stage

The second stage further filtered the 166 structures to 91 potentially catalytically active trypsins using EnzyMM to confirm catalytic residues presence. Four filters were then applied: solubility ≥ 0.69, *T*_m_ ≥ 55, *T*_opt_ [30:45], and pH_opt_ [7:10]. 24 trypsins passed these specified filters.

#### 2.2.3 Scoring

Using the predicted values from NetSolP - 1.0, pHoptNN, and Seq2Topt, a score on a 0–1 scale was assigned to represent the performance of an enzyme compared to other enzymes. For values filtered by cutoffs, scores were normalized to 0–1 using min-max scaling:


(1) $$\mathrm{score}(v)=\max\left(0,\min\left(1,\frac{v-\;l}{h-\;l}\right)\right)$$

As shown in Equation (1), the enzyme with a predicted value *v* that equalled the lowest value observed *l*, had a 0 score. Similarly, the enzyme with the highest predicted value observed *h*, scored 1. The rest had scores within this range.

For interval filtered values, scores were calculated via a triangular function centred on the interval midpoint.


(2) score(v)=max(0, min(1, 1-|v-m|/w))

Equation (2) shows that the score rises linearly with decreasing half-width *w*, a dependent parameter equals *(h-l)/2*. Predicted values at both edges received a score of 0, while the score was 1 at the midpoint *m*. The Enzymes in the filtered set had a score of 0–1 for each value of the four predicted properties. The composite score to assess their performance across these properties simultaneously was computed by taking the average of the four scores.

The previous two equations rank candidates within the enzyme set that already passed user’s filters. The mean is taken over only the properties that the user specifies, for example solubility and melting temperature, the choice of filters determines the properties that contribute to the ranking to enable users to limit to certain attributes.

#### 2.2.4 Clades and representatives

**Figure 1:**
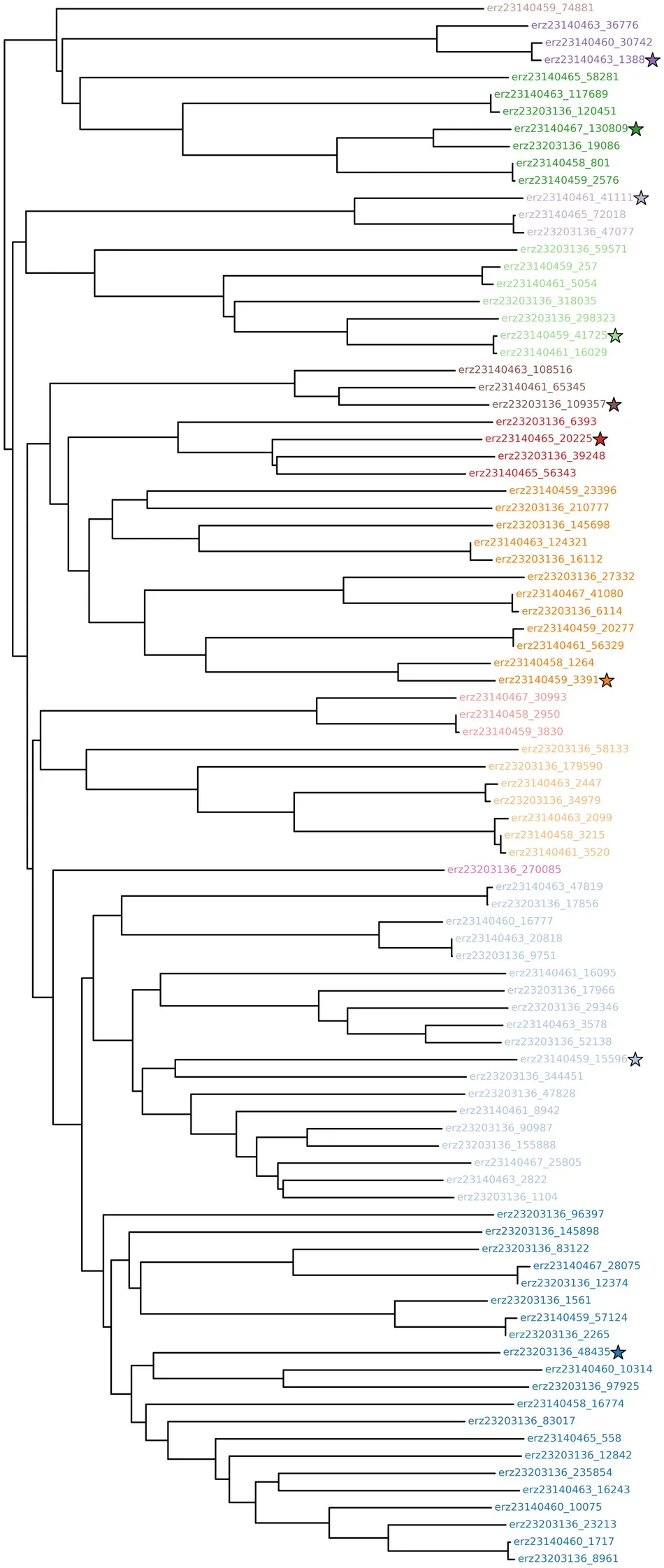
Generated tree with color-highlighted clades, clades representatives are marked using stars.

Clades were defined by partitioning the generated tree using a distance threshold *T*. The methods computed the subtree depth for every internal node as the longest path from that node to any of its leaves. Every subtree whose depth was ≤ *T*, was emitted as a single clade. *T* corresponding to the user-specified clade number was determined by binary search over *T* ∈ [0, *D*], where *D* is the depth of the whole tree. At each iteration the number of clades was checked. If it exceeded that provided by the user, the lower bound was raised, otherwise the upper bound was lowered until reaching the user specified number of clades (Fig. 1).

## 3 Results

Figure 1 shows the tree generated for the 91 potentially catalytically active trypsins, partitioned into 13 clades that we specified for diversity. Four clades had no single enzyme that passed all filters, hence no enzyme was eligible to be a clade representative. Using this tree, we identified nine trypsins from different clades with the best overall predicted values according to user supplied filtering criteria. The output results of the test run are available at Supplementary data S1.

## 4 Conclusion

EnzymeSifter enables users to extensively search metagenomes identifying candidate enzymes with *in silico* predicted properties passing the user defined filters. Enzymes are given scores to represent their predicted performance and are clustered into clades to mark the best performer of each clade. This enables users to vastly reduce testable enzyme sequence space and subsequently prioritize these non-redundant best in clade enzymes for experimental validation. We note that EnzymeSifter inherits the limitations of its integrated tools. The composite score is controlled by user input and the candidates it selects represent hypotheses for experimental testing. Future work will extend the tool to incorporate further predicted properties.

## Supplementary Material

vbag262_Supplementary_Data

## Data Availability

EnzymeSifter source code is freely available and released under an MIT licence. It is available at https://github.com/Bashton-Lab/EnzymeSifter. Sequences used in the test run are publicly available at https://doi.org/10.5281/zenodo.2,07,20,235 this also contains the AlphaFold predicted structures. AlphaFold Server output is used in accordance with the AlphaFold Server Output Terms of Use.
