# Supplementary material for: EnzymeSifter: a tool for discovery of industrial enzymes from metagenomes
Source: Bioinform Adv. 2026 Sep 3;6(1):vbag262. doi: 10.1093/bioadv/vbag262 (PMC13597120; doi:10.1093/bioadv/vbag262)
Supplement: Supplementary file 1 [file vbag262_supplementary_data.zip › Supplementary_data/Figure S1.pdf]

```
                ┌────────────────────────────────┐
                │          Input FASTA           │
                └───────────────┬────────────────┘
                                │
        ╔═══════════════════════▼════════════════════════╗
        ║                    STAGE 1                      ║
        ║  motif → Pfam (HMMER) → EC (CLEAN) → MMseqs2    ║
        ╚═══════════════════════╤════════════════════════╝
                                │
                ┌───────────────▼────────────────┐
                │ data/stage1/nonredundant.fasta │
                └───────────────┬────────────────┘
                                │
        ╔═══════════════════════▼════════════════════════╗
        ║              predict 3D structures              ║
        ║                done by the user                 ║
        ╚═══════════════════════╤════════════════════════╝
                                │
                ┌───────────────▼────────────────┐
                │      directory of .pdb files    │
                └───────────────┬────────────────┘
                                │
╔═══════════════════════════════▼══════════════════════════════════════════╗
║                               STAGE 2                                     ║
║   EnzyMM ── filter to enzymatic hits ──┬── NetSolP  (solubility, usability) ║
║                                        ├── pHoptNN  (optimum pH)            ║
║                                        ├── Seq2Topt (optimum T)             ║
║                                        ├── Seq2Tm   (melting T)             ║
║                                        └── MUSCLE ─► NJ tree ─► clades ──►  ║
║                                                   representatives          ║
╚═══════════════════════════════╤══════════════════════════════════════════╝
                                │
                ┌───────────────▼────────────────┐
                │  predictions_output/*.tsv      │
                │  data/trees/nj_tree_clades.png │
                └────────────────────────────────┘
```
