# Supplementary material for: EnzymeSifter: a tool for discovery of industrial enzymes from metagenomes
Source: Bioinform Adv. 2026 Sep 3;6(1):vbag262. doi: 10.1093/bioadv/vbag262 (PMC13597120; doi:10.1093/bioadv/vbag262)
Supplement: Supplementary file 1 [file vbag262_supplementary_data.zip › Supplementary_data/Note S1.pdf]

Comparison of EnzymeSifter integrated predictors against direct alternatives

This document lists the performance of integrated tools in EnzymeSifter over others that predict the same property. Each table compares an integrated tool against other tools using values reported from the tools' own developers on a benchmark originally carried out by the developers of the original tool in their respective publications. In each case we have used the most recent tool which is shown to outperform all previous tools within its own publication.

Some tools that can predict certain values used in EnzymeSifter were not considered for the comparison if these tools are web-only tools with no source code available, and or cannot be wrapped easily into EnzymeSifter for direct installation, or if they are specific to certain enzyme classes rather than general tools.

Seq2Topt

The four tools that predict optimal temperature (T_{opt}) were evaluated by Qiu et al. (2025) on the hold-out test set (*n*=291) obtained from a TOMER/BRENDA dataset of enzymes with experimentally determined T_{opt}. They reported the root mean squared error (RMSE) and coefficient of determination (R2).

Tool	Input required	R2	RMSE (°C), T_{opt} > 60 °C
Seq2Topt	Sequence	0.57	17.35
TOMER	Sequence + OGT	0.55	9.85
DeepET	Sequence + OGT	0.49	11.74
Preoptem	Sequence	0.35	21.92

R2 of Seq2Topt is the highest among the four tools. TOMER and DeepET have lower RMSE values in the T_{opt} > 60 °C range, but these require the organism optimal growth temperature (OGT) which will not be available in the case of a metagenomics dataset. Among sequence only tools, Seq2Topt is thus the top performer without the requirement for OGT.

Moreover, a model called Seq2Tm was developed from the architecture of Seq2Topt to predict the melting temperature (T_m). The authors benchmarked Seq2Tm against DeepTM on the same DeepTM test set (1550 entries, T ≥ 50 °C). Although Seq2Tm had slightly higher RMSE (7.57 °C) compared to (7.50 °C) by DeepTM, the later requires organism OGT.

pHoptNN

All models for optimum pH (pH_{opt}) prediction were evaluated by SinhaRoy et al. (2026) on the held-out test set, which is 20% of the EnzyBase12k dataset of 11,615 enzymes with experimentally determined pH optima and high-quality three-dimensional structures. They reported RMSE, MSE, R2, Pearson's r and Spearman's ρ. pHoptNN requires the three-dimensional structure.

Model	RMSE	MSE	R2	Pearson's r	Spearman's ρ
pHoptNN	0.588	0.346	0.79	0.89	0.78

Model	RMSE	MSE	R2	Pearson's r	Spearman's ρ
GCN	0.610	0.372	0.79	0.89	0.77
MPNN + Regressor	0.642	0.412	0.66	0.81	0.72
OpHReda	0.774	0.599	0.62	0.80	0.71
EpHod	0.866	0.750	0.53	0.73	0.63

pHoptNN gives the lowest RMSE and MSE and the highest correlations among the compared models in part by using structure as input. The improvement over the sequence-based approaches (OpHReda and EpHod) is large and since our users are already providing predicted PDB structures for stage 2 here we have chosen to use a structure-based method with pHoptNN.

NetSolP

All tools were evaluated by Thumuluri et al. (2022) using multiple datasets to evaluate solubility prediction accuracy. The reported values for Price independent test set (1,323 sequences) are shown below with accuracy (ACC), precision (PRE), Matthew's correlation coefficient (MCC), and a threshold-independent metric, area under the Receiver Operating Characteristic curve (AUC).

Method	ACC	PRE	MCC	AUC
NetSolP	0.728	0.773	0.402	0.760
NetSolP-D	0.723	0.769	0.391	0.756
SoluProt	0.624	0.704	0.187	0.634
Parsnip	0.558	0.786	0.221	0.663
Camsol	0.570	0.751	0.199	0.646
DeepSol-S2	0.450	0.764	0.117	0.595
Protein-Sol	0.641	0.694	0.190	0.679
SWI	0.680	0.712	0.269	0.690
ESM12-F	0.698	0.743	0.328	0.732
ESM1b-F	0.723	0.764	0.386	0.761
ProtT5-P	0.702	0.714	0.314	0.733
ESM-MSA-P	0.712	0.715	0.340	0.745

Tools were also compared by Thumuluri et al. (2022) on usability using Price usability set (7,259 sequences).

Method	ACC	PRE	MCC	AUC
NetSolP	0.65 ± 0.02	0.65 ± 0.04	0.30 ± 0.04	0.70 ± 0.01
SoluProt (retrained)	0.63 ± 0.02	0.63 ± 0.03	0.26 ± 0.03	0.63 ± 0.02
SoluProt	0.62 ± 0.01	0.60 ± 0.02	0.24 ± 0.02	0.67 ± 0.02
Parsnip	0.61 ± 0.01	0.66 ± 0.02	0.22 ± 0.01	0.66 ± 0.01
Camsol	0.55 ± 0.02	0.63 ± 0.12	0.13 ± 0.07	0.62 ± 0.05
DeepSol-S2	0.54 ± 0.02	0.59 ± 0.06	0.09 ± 0.05	0.57 ± 0.02
Protein-Sol	0.55 ± 0.02	0.53 ± 0.02	0.13 ± 0.04	0.60 ± 0.02
SWI	0.58 ± 0.01	0.54 ± 0.01	0.20 ± 0.02	0.64 ± 0.02

Method	ACC	PRE	MCC	AUC
ESM12-F	0.64 ± 0.01	0.63 ± 0.01	0.29 ± 0.03	0.71 ± 0.01
ESM1b-F	0.65 ± 0.01	0.64 ± 0.03	0.30 ± 0.03	0.71 ± 0.01

Here NetSolP and NetSolP-D are clearly top performers so were chosen. The authors reported that NetSolP-D takes about 3s per sequence. They also reported that Camsol, ProteinSol and SWI scale well, but the previous 2 tables show that NetSolP outperforms them.

CLEAN

All values below for EC number prediction were reported by Yu et al. (2023) on several datasets, they reported: precision, recall and F1 score (harmonic mean of precision and recall).

New-392 dataset: 392 enzyme sequences covering 177 different EC numbers, taken from Swiss-Prot entries released after CLEAN was trained.

Tool	Precision	Recall	F1
CLEAN	0.5965	0.4811	0.4988
ProteInfer	0.4088	0.2843	0.3086
DeepEC	0.2976	0.2167	0.2297
ECPred	0.1178	0.0954	0.1000

Price-149 dataset: experimentally validated sequences that were curated by ProteInfer as a challenging benchmark.

Tool	Precision	Recall	F1
CLEAN	0.5844	0.4671	0.4947
BLASTp	0.5083	0.3750	0.3852
ProteInfer	0.2434	0.1382	0.1662
DeepEC	0.1184	0.0724	0.0846
DEEPre	0.0415	0.0403	0.0386
ECPred	0.0197	0.0197	0.0197

Dataset of underrepresented EC numbers: more than 3,000 sequences covering more than 1,000 EC numbers, where each EC number occurs no more than five times. ProteInfer and DeepEC were evaluated using their released pretrained models, so this dataset had already been seen during their training, which gives them an advantage that CLEAN did not have.

Tool	Precision	Recall	F1
CLEAN	0.834	0.813	0.817
ProteInfer	0.848	0.751	0.782
DeepEC	0.712	0.586	0.625

CLEAN has the highest F1 score on all three datasets. On the underrepresented EC numbers, ProteInfer reaches a slightly higher precision but a lower recall, so CLEAN still leads on F1 despite

ProteInfer having seen this dataset during training. CLEAN was thus chosen for EC number prediction.

References

Qiu, S., Hu, B., Zhao, J., Xu, W., & Yang, A. (2025). Seq2Topt: a sequence-based deep learning predictor of enzyme optimal temperature. *Briefings in Bioinformatics*, *26*(2), bbaf114.

SinhaRoy, R., Clauss, C., Ivanikov, I., & Kuenze, G. (2026). Predicting Enzyme pH Optima from Structure Using Equivariant Graph Neural Networks. *bioRxiv*, 2026-01.

Thumuluri, V., Martiny, H. M., Almagro Armenteros, J. J., Salomon, J., Nielsen, H., & Johansen, A. R. (2022). NetSolP: predicting protein solubility in Escherichia coli using language models. *Bioinformatics*, *38*(4), 941-946.

Yu, T., Cui, H., Li, J. C., Luo, Y., Jiang, G., & Zhao, H. (2023). Enzyme function prediction using contrastive learning. *Science*, *379*(6639), 1358-1363.
